# Whole‐body senescent cell clearance alleviates age‐related brain inflammation and cognitive impairment in mice

**DOI:** 10.1111/acel.13296

**Published:** 2021-01-20

**Authors:** Mikolaj Ogrodnik, Shane A. Evans, Edward Fielder, Stella Victorelli, Patrick Kruger, Hanna Salmonowicz, Bettina M. Weigand, Ayush D. Patel, Tamar Pirtskhalava, Christine L. Inman, Kurt O. Johnson, Stephanie L. Dickinson, Azucena Rocha, Marissa J. Schafer, Yi Zhu, David B. Allison, Thomas von Zglinicki, Nathan K. LeBrasseur, Tamar Tchkonia, Nicola Neretti, João F. Passos, James L. Kirkland, Diana Jurk

**Affiliations:** ^1^ Department of Physiology and Biomedical Engineering Mayo Clinic Rochester MN USA; ^2^ Robert and Arlene Kogod Center on Aging Mayo Clinic Rochester MN USA; ^3^ Department of Molecular Biology, Cell Biology and Biochemistry Brown University Providence RI USA; ^4^ Biostatistics Consulting Center School of Public Health‐Bloomington Indiana University Bloomington IN USA; ^5^ Faculty of Medical Sciences Biosciences Institute Campus for Ageing and Vitality Newcastle University Newcastle upon Tyne UK; ^6^ Present address: Arts and Sciences Faculty, Molecular Biology and Genetics Near East University Mersin Turkey

**Keywords:** aging, brain, cognition, memory, neurodegeneration, SASP, senescence, senolytic, telomeres

## Abstract

Cellular senescence is characterized by an irreversible cell cycle arrest and a pro‐inflammatory senescence‐associated secretory phenotype (SASP), which is a major contributor to aging and age‐related diseases. Clearance of senescent cells has been shown to improve brain function in mouse models of neurodegenerative diseases. However, it is still unknown whether senescent cell clearance alleviates cognitive dysfunction during the aging process. To investigate this, we first conducted single‐nuclei and single‐cell RNA‐seq in the hippocampus from young and aged mice. We observed an age‐dependent increase in p16^Ink4a^ senescent cells, which was more pronounced in microglia and oligodendrocyte progenitor cells and characterized by a SASP. We then aged *INK*‐*ATTAC* mice, in which p16^Ink4a^‐positive senescent cells can be genetically eliminated upon treatment with the drug AP20187 and treated them either with AP20187 or with the senolytic cocktail Dasatinib and Quercetin. We observed that both strategies resulted in a decrease in p16^Ink4a^ exclusively in the microglial population, resulting in reduced microglial activation and reduced expression of SASP factors. Importantly, both approaches significantly improved cognitive function in aged mice. Our data provide proof‐of‐concept for senolytic interventions' being a potential therapeutic avenue for alleviating age‐associated cognitive impairment.

## Graphical abstract

Senescence is a major contributor to aging and age‐related diseases. However, it is still unknown whether senolytics impact on cognitive function during the aging process. We found that both pharmacogenetic clearance of p16Ink4a senescent cells or treatment with senolytic cocktail Dasatinib and Quercetin, reduced senescent microglia in the hippocampus and improved cognitive function in aged mice.

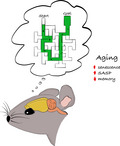

## 1 INTRODUCTION

The aging process is characterized by a progressive decline in memory, attentional control, orientation, and cognition (Camandola & Mattson, 2017; Hof & Morrison, 2004). Furthermore, aging is a major risk factor for development of several neurodegenerative disorders such as amyotrophic lateral sclerosis (ALS), primary progressive multiple sclerosis (PPMS), Alzheimer's disease (AD), and Parkinson's disease (PD) (Hou et al., 2019).

Cellular senescence is a process characterized by an irreversible cell cycle arrest that can be induced by a variety of stresses, including oxidative stress, mitochondrial dysfunction, and telomere dysfunction, among others (Baker et al., 2016; Correia‐Melo et al., 2016; Hewitt et al., 2012; Ogrodnik et al., 2019). Senescent cells have been shown to accumulate in a variety of tissues with aging and at the etiological sites of chronic diseases (McHugh & Gil, 2018).

Senescent cells are thought to exert their detrimental effects with aging and in age‐related diseases partially via the senescence‐associated secretory phenotype (SASP) (Coppé et al., 2008), which involves increased secretion of a number of pro‐inflammatory cytokines, chemokines, growth factors, and extracellular matrix proteases. The SASP can have beneficial effects, such as orchestrating the clearance of senescent cells (Ovadya et al., 2018; Xue et al., 2007). However, chronic exposure to the SASP is thought to contribute to the spreading of senescence to otherwise healthy tissues (Acosta et al., 2013; Nelson et al., 2012; Xu et al., 2018).

Senescent cells have been shown to accumulate in the brain with aging. Senescent cell markers have been found in various brain cell types such as neurons, astrocytes, oligodendrocyte progenitor cells, microglia, ependymal cells, and endothelial cells (Bussian et al., 2018; Chinta et al., 2018; Jurk et al., 2012; Musi et al., 2018; Ogrodnik et al., 2018; Zhang et al., 2019). Furthermore, recent studies have shown that clearance of senescent cells had beneficial effects in mouse models of AD and tau‐dependent neurodegenerative diseases (Bussian et al., 2018; Musi et al., 2018; Zhang et al., 2019), PD (Chinta et al., 2018), PPMS (Nicaise et al., 2019), and ALS (Trias et al., 2019). However, currently it is not known if presence of senescent cells influences the progression of brain aging and age‐associated cognitive impairment.

Here, we utilized the *INK*‐*ATTAC* transgenic mouse model, in which apoptosis of highly p16^Ink4a^‐expressing cells can be induced upon administration of the drug AP20187 (AP) (Baker et al., 2011). Of note, not every cell with high p16^Ink4a^ expression is senescent and not every senescent cell has high levels of p16^Ink4a^ expression. The second strategy we used is senolytic drug treatment with the combination of Dasatinib and Quercetin (D + Q), which were shown to clear senescent cells in vitro (Aguayo‐Mazzucato et al., 2019; Zhu et al., 2015), in vivo in peripheral organs (Aguayo‐Mazzucato et al., 2019; Ogrodnik et al., 2017; Xu et al., 2018; Zhu et al., 2015), and in the brain (Ogrodnik et al., 2018; Zhang et al., 2019). These drugs act by transiently disabling the Senescent Cell Anti‐apoptotic Pathways (SCAPs) that defend senescent cells from apoptosis; they do not act by killing cells based only on expression of p16^Ink4a^. The fundamental difference between both approaches is the fact that, in contrast to the *INK*‐*ATTAC* model which targets specifically highly p16^Ink4a^‐expressing cells, the D + Q senolytic cocktail does not target a specific molecule or biochemical pathway. Indeed, we have previously found that D targets tyrosine kinases, while flavonoid Q targets BCL‐2 family members as well as HIF‐1α and particular nodes in PI3‐kinase pathways (Zhu et al., 2015). Because different senescent cell types utilize diverse SCAPs to defend themselves against their own pro‐apoptotic microenvironment, we predict that strategies targeting multiple SCAPs will be more effective at removing senescent cells than drugs that have a single‐molecular target.

We observed by single‐nucleus and single‐cell RNA‐seq that p16^Ink4a^ positive cells increase in the hippocampus of aged mice in different cell populations; however, p16^Ink4a^ is more abundant in microglia and oligodendrocyte progenitor cells. Intermittent, two‐month long treatment of aged *INK*‐*ATTAC* mice with AP or senolytics resulted in a significant attenuation of age‐associated cognitive dysfunction measured by the Stone T‐Maze. Moreover, we observed a reduction of p16^Ink4a^ exclusively in the microglial population in the CA3 region of the hippocampus in both AP‐ and D + Q‐treated mice. Finally, we detected a mild but significant decrease in markers of inflammation, such as expression of pro‐inflammatory molecules, microglial activation, and infiltration of CD3‐positive T cells. Together, these data suggest that clearance of senescent cells is a viable therapeutic strategy to counteract age‐related cognitive decline.

## 2 RESULTS

### 2.1 Senescent cells accumulate in the brain during aging and exhibit changes in secretory phenotype

**FIGURE 1:**
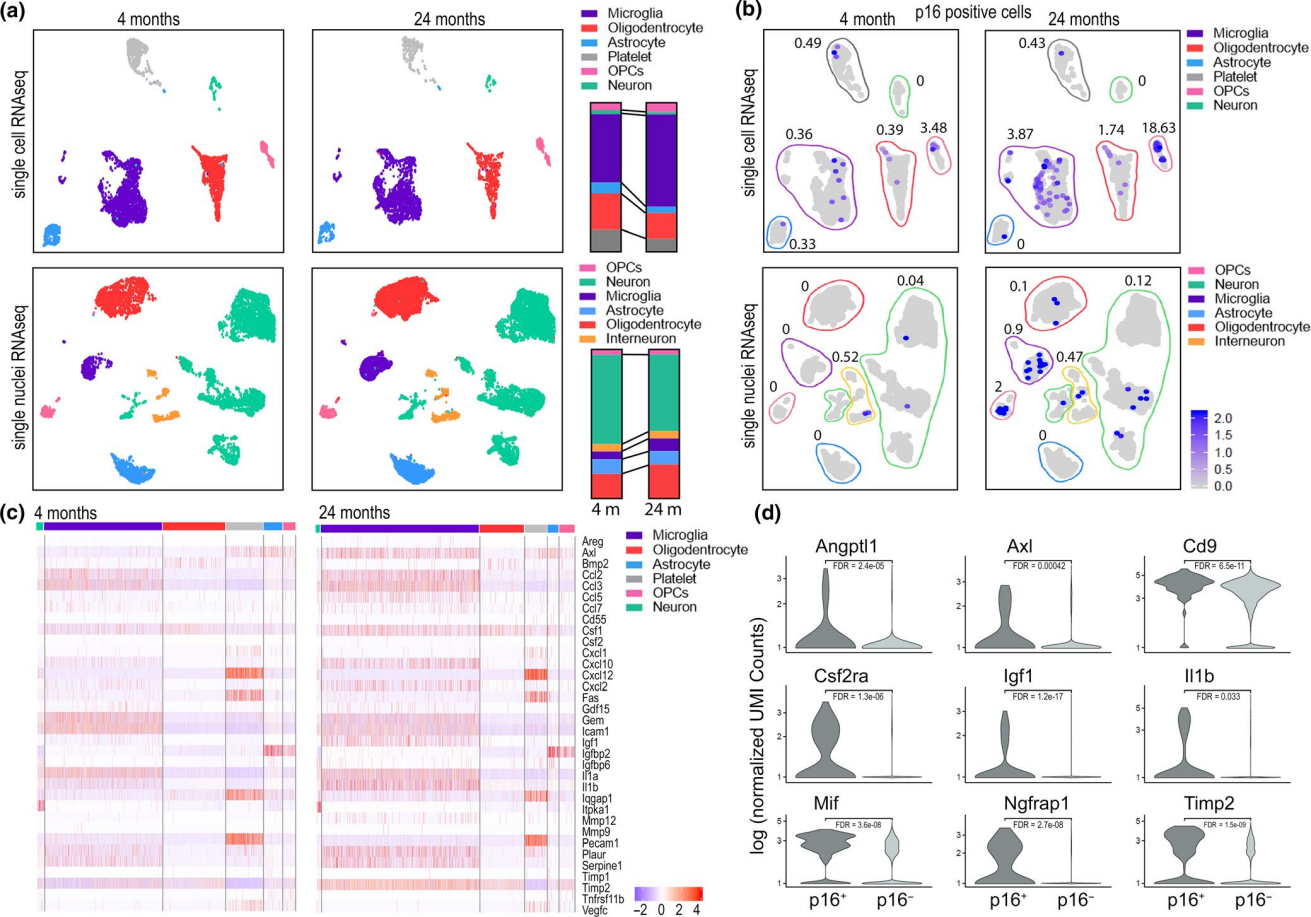
Senescent cells accumulate in hippocampus during aging. Hippocampal tissues were isolated from young (4 months) and old (24 months) mice. Single‐cell (sc) or single‐nucleus (sn) suspensions were prepared from hippocampal tissue and used for RNA‐seq. (a) UMAP embeddings of sc‐RNA‐seq (top) or sn‐RNA‐seq (bottom) were investigated in our datasets. Colors represent cell type annotations. Bar graphs on the right show changes in fractions of cell populations isolated from young and old mice. (b) Matching UMAP embeddings with expression of p16^Ink4a^ for sc‐RNA‐seq (top) or sn‐RNA‐seq (bottom) datasets. Color frames represent cell type annotation as above. % of cells positive for high levels of p16^Ink4a^ is shown next to each cell population. (c) Heatmap is showing expression of cytokines in various types of brain cells. Bars above the heatmap indicate color‐matched cell types consistent with graphs above. (d) Violin plots show expression of cytokines in cells showing high or no expression of p16^Ink4a^. Differential expression was carried out with the Seurat and MAST packages for single‐cell analysis. Multiple testing correction was conducted by computing a false discovery rate (bonferroni correction) using all genes in the dataset. The majority of significantly (fdr <0.05) differentially expressed SASP genes were upregulated in Cdkn2a + cells. Total of 4 mice per condition (young, old) are analyzed, while 2 mice are pooled for each experiment

Previous data have indicated that cell senescence is a feature of brain pathology and aging (Bussian et al., 2018; Chinta et al., 2018; Fielder et al., 2020; Jurk et al., 2012; Musi et al., 2018; Nicaise et al., 2019; Trias et al., 2019; Zhang et al., 2019). However, the identity of senescent cells in the brain remains elusive as majority of studies utilized semi‐quantitative techniques (which rely on tissue homogenization) or immunohistochemical techniques that do not offer a comprehensive analysis of senescence in different cell populations. For these reasons, in this study we used single‐cell RNA sequencing (sc‐RNA‐seq) and single‐nucleus RNA sequencing (sn‐RNA‐seq) to profile and compare the cellular composition and transcriptomes of 4 young (4 months) and 4 old (24 months) mouse hippocampi, a region that plays a key role in memory formation (Figure 1, Figure S1). The dissociation and processing of mammalian adult brain tissue is challenging due to its complexity and can result in reduced yield of certain cell types, such as neurons. To address this issue and to generate datasets consisting of a broad range of brain cell types, we utilized two separate dissociation approaches prior to RNA sequencing. Firstly, we generated single‐cell suspensions from the hippocampus, which we found by RNA‐seq analysis to be enriched in glial cells, predominantly microglia, oligodendrocytes, and astrocytes (Figure 1a and Figure S1A top panel). We pooled 2 hippocampi per experiment and used a total of 4 mice per group (Figure S1A). Second, we generated single‐nucleus suspensions by isolating nuclei of individual brain cells and found that, using this method, we significantly enriched the population of neurons and interneurons in the suspension, with a smaller fraction of glial cells present (Figure 1a and Figure S1A bottom panel).

Next, we assessed what populations of brain cells acquire markers of senescence. Quantification of p16^Ink4a^ expression in the sc‐RNA‐seq dataset showed an age‐related increase in the number of p16^Ink4a^‐positive cells, with the most prominent increase in the populations of microglia and oligodendrocyte progenitor cells (OPCs) (Figure 1b top panel). Of note, the highest percent increase in p16^Ink4a^‐positive cells was observed in the OPCs population, while the highest total number of p16^Ink4a^‐positive cells was observed in the microglial population (Figure 1b top panel). sn‐RNA‐seq showed that, in addition to accumulation of p16^Ink4a^‐positive microglia and OPCs, neurons and interneurons also show an age‐related increase in the fraction of p16^Ink4a^‐positive nuclei (Figure 1b bottom panel). However, the fraction of the population with high p16^Ink4a^ expression was much lower in the sn‐RNA‐seq than the sc‐RNA‐seq dataset (Figure 1b), suggesting that the majority of p16^Ink4a^ mRNA is in the cytoplasm of brain cells. Similar changes were observed in the frequency of p21‐positive brain cells, including an age‐related increase in the population of microglia, OPCs, and oligodendrocytes (Figure S1B). Of note and in contrast to p16^Ink4a^, p21 was also increased in aged astrocytes (Figure S1B).

Leveraging the sn‐RNA‐seq and sc‐RNA‐seq datasets, we analyzed age‐related changes in expression levels of cytokines and growth factors in the abovementioned cell populations. We found that hippocampal aging is characterized by increases in the fraction of cells positive for Axl, Csf1, Csf2ra, Igf1, Il‐1β, Mif, Plaur and Timp2, among others (Figure 1c,d). Importantly, the cell population that showed the highest expression of the listed cytokines is microglia (Figure 1c). It should be noted that the sc‐RNA‐seq dataset was more sensitive for detecting age‐related changes in cytokine mRNA than the sn‐RNA‐seq dataset (Figure S1C). Finally, assessment of expression of cytokines in p16^Ink4a^‐positive populations revealed that expression of p16^Ink4a^ positively correlates with higher expression of pro‐inflammatory molecules in the sc‐RNA‐seq dataset (Figure 1d) and these changes were the most pronounced in the microglial population (Figure S1D,C). Overall, these observations led us to hypothesize that age‐related accumulation of senescent cells impacts hippocampal function and may contribute to cognitive decline.

### 2.2 Clearance of senescent cells alleviates age‐associated cognitive impairment

**FIGURE 2:**
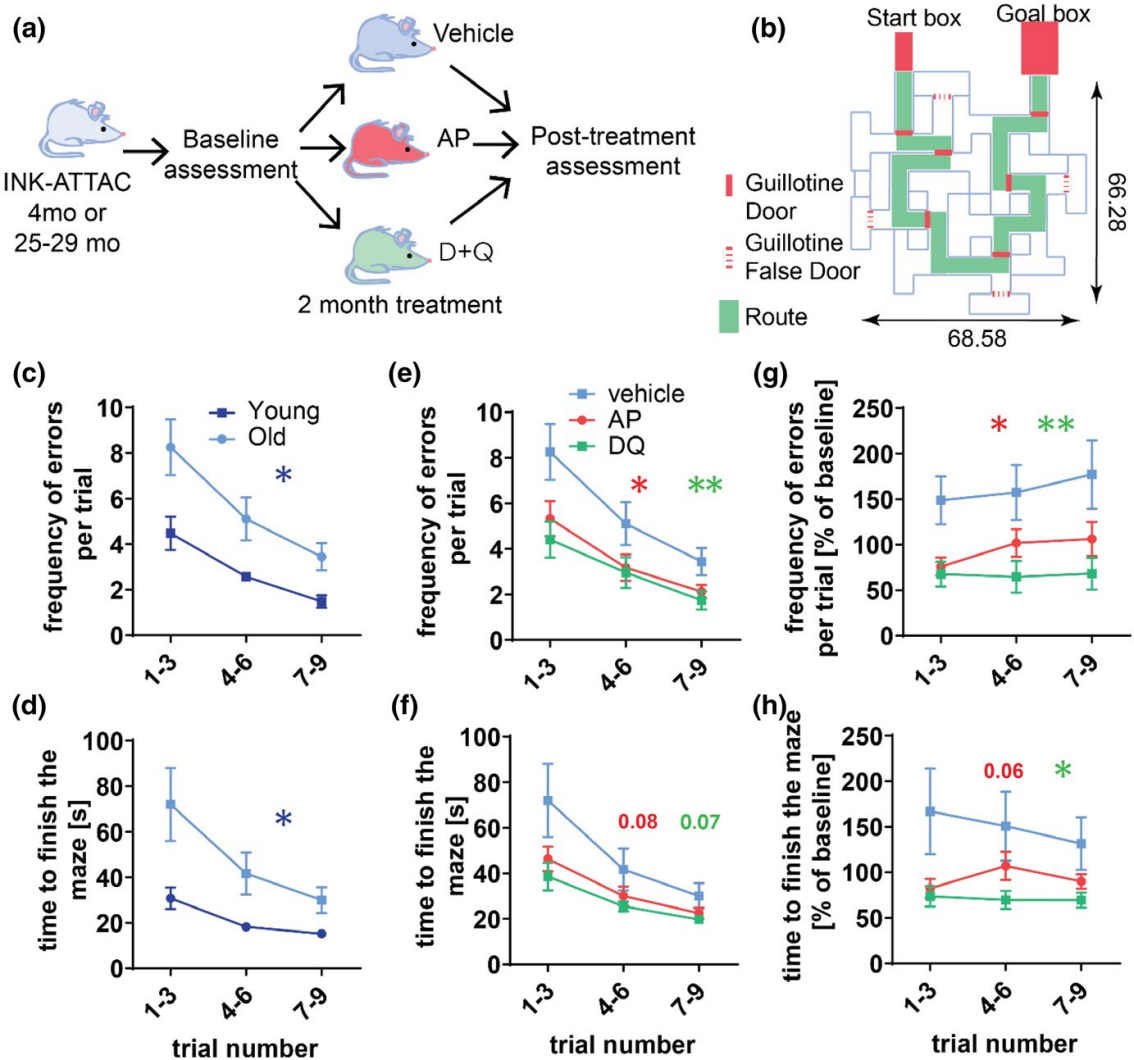
Whole‐body senescent cell clearance alleviates age‐induced spatial memory dysfunction. (a) 4‐month‐old and 25–29‐month‐old mice were split into three groups and assigned to vehicle (v), Dasatinib and Quercetin (D + Q), or AP20187 (AP) treatments. Animals were matched by baseline parameters: body mass, body composition, Rotarod performance, and cognition. After 2 months of drugs or vehicle, mice were tested again (post‐treatment assessment) for changes in body parameters, cognition, and Rotarod performance. Shortly after the post‐treatment assessments, mice were sacrificed. (b) Schematics of the Stone T‐maze are presented. All of the numbers designating dimensions indicate length in centimeters. The parameters of the spatial memory (c), the frequency of errors, and (d) the time to finish the test were assessed in young and old *INK*‐*ATTAC* mice using the Stone T‐maze. As the effect of AP, D + Q, or vehicle treatment on memory functional parameters: (e) the frequency of errors and (f) the time to finish the test were assessed in old mice. Post‐treatment results: (g) frequencies of errors and (h) the time to finish the maze were normalized to baseline and analyzed. All data are mean ± SEM with *n* = 9–11 for old mice, *n* = 7–8 for young mice. *p < 0.05, **p < 0.01

To investigate the impact of senescent cell elimination on cognition, we utilized cohorts of 4‐month‐old and 25–29‐month‐old *INK*‐*ATTAC* male mice, which were intermittently administered AP20187 (AP) or a senolytic drug cocktail (D + Q) for 2 months (Figure 2a). During the study we lost several aged mice (6 vehicle‐treated, 1 AP‐treated, and 3 DQ‐treated) and 1 young mouse, but we observed no significant differences in survival among AP‐treated old mice (*p* = 0.06) or D + Q‐treated old mice (*p* = 0.61) (Figure S2A,B). To avoid the confounding by this survival effect, all phenotypic assessments were performed before (baseline) and after treatment (post‐treatment).

Vision and sense of smell deteriorate in C57Bl/6 mice with aging (Patel & Larson, 2009; Pettan‐Brewer & Treuting, 2011), which can be a confounding factor in memory and cognition assessment. Thus, we used a modified Stone T‐maze (Pistell & Ingram, 2010; Pistell et al., 2012) that utilizes escape from shallow water as motivation (Figure 2b). Importantly, this test relies on the brain's navigational system. Therefore, it is not impacted by sight or olfaction and has been shown to be effective in evaluating learning and memory ability in aging C57Bl/6 mice (Pistell et al., 2012). Using the Stone T‐maze to assess memory function in *INK*‐*ATTAC* mice, we confirmed that aged animals make significantly more errors before escaping from the maze (*p* = 0.0137) (Figure 2c) and need a longer time to complete the test (*p* = 0.0341) (Figure 2d), both compared to the younger animals. We found that neither AP‐ nor senolytic‐treated aged mice showed a significant reduction (AP *p* = 0.08, D + Q *p* = 0.07) in the amount of time needed to finish the maze compared to vehicle controls (Figure 2f). However, both AP and D + Q treatment resulted in a significant reduction in the frequency of errors (AP *p* = 0.038, D + Q *p* = 0.019) made by aged mice (Figure 2e; Appendix Video [Link], [Link], [Link]). Importantly, the results were still significant after the post‐treatment data were normalized to the baseline measurements (Figure 2g,h), suggesting that neither differences in body composition nor mortality affected the assessment. Finally, neither AP nor D + Q treatment impacted the time young mice needed to finishing the test (Figure S2C) or errors performed during the test (Figure S2D), indicating that the treatments only have a significant impact during aging. These data show that whole‐body clearance of highly p16^Ink4a^‐expressing or senescent cells alleviates age‐associated cognitive impairment.

Treatment with either AP or senolytics did not significantly change body weight in aged mice (Figure S2E,F). During the treatment period, we observed that aged vehicle‐treated mice had a decrease in total body fat (Figure S2G). Interestingly, we found that mice treated with AP but not D + Q retained significantly more fat than their vehicle‐treated littermates (Figure S2H), which is consistent with previously published results (Baker et al., 2016; Xu et al., 2015). To assess neuromuscular function, we performed the Rotarod test. Consistent with previous data, we found that aged mice perform less well than their young counterparts and that AP treatment significantly improves neuromuscular function (Figure S2I). However, the post‐treatment results were no longer significant when the data are normalized to baseline measurements (Figure S2J). This is likely a result of a higher mortality rate in low‐weight animals in the vehicle‐treated group, as we found that Rotarod performance is inversely related to weight (Figure S2K). Using the open field test, we found that neither aging nor treatment significantly affected activity in our mouse cohort (Figure S2L,M).

### 2.3 Senotherapies target p16^Ink4a^‐positive microglia and reduce markers of neuronal senescence in the CA3 region of the hippocampus

The hippocampus is one of the core regions involved in memory function and cognition and undergoes progressive deterioration with aging. Importantly, sub‐regions of the hippocampus age differently, with the sub‐regions, *Cornu amonis* 3 (CA3) and *Cornu amonis* 1 (CA1), being more robustly affected (Oh et al., 2016).

RT‐PCR performed on isolated murine hippocampi (Figure S3A) showed that aged *INK*‐*ATTAC* mice exhibit increased expression of p16^Ink4a^, however, AP treatment has minimal effects in terms of reducing expression of p16^Ink4a^. We reasoned this could be due to the fact that only a small sub‐set of hippocampal cells show high p16^Ink4a^ expression and are eliminated following AP treatment and that RT‐PCR in homogenized tissues may not be sensitive enough to pick up these differences.

**FIGURE 3:**
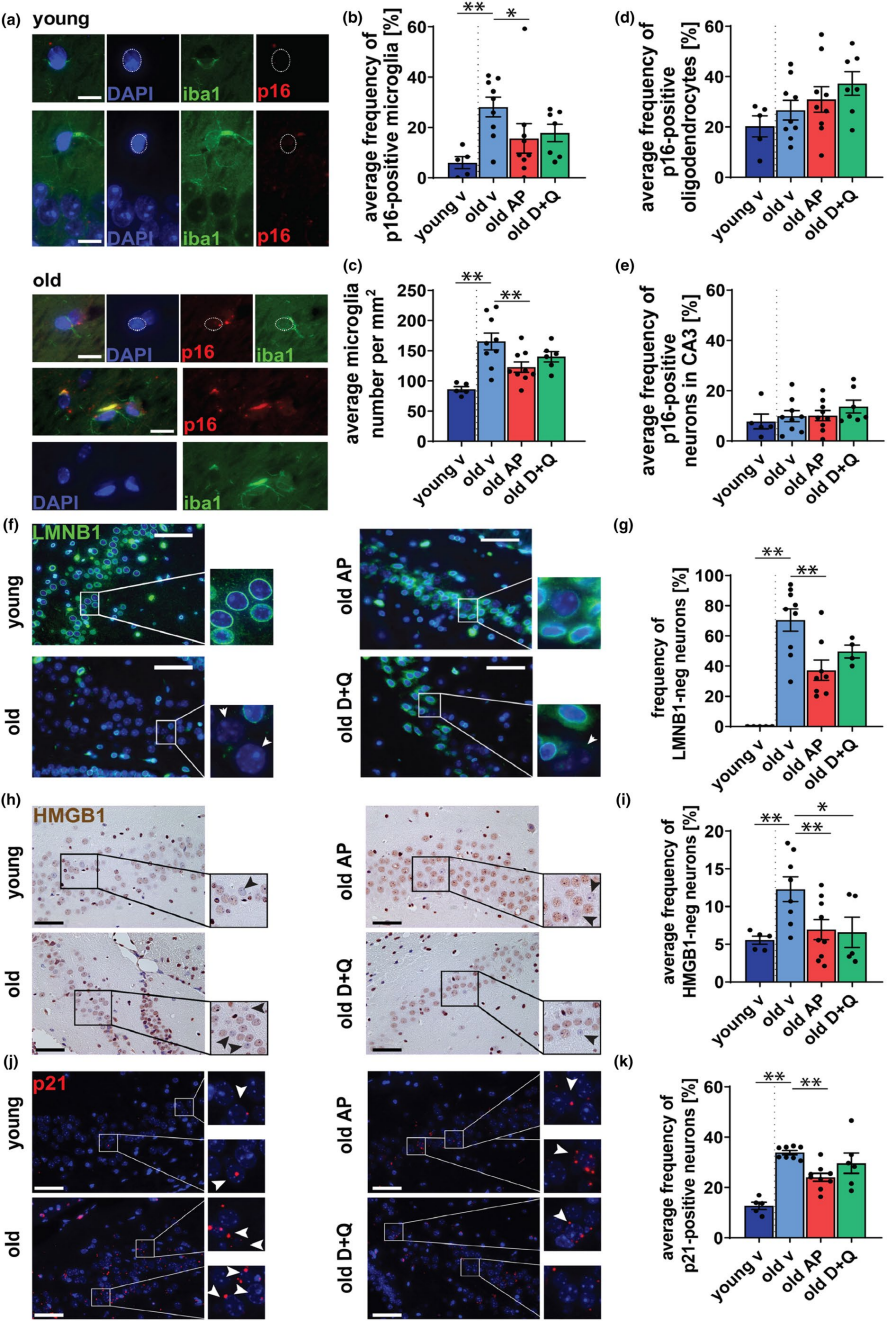
Clearance of senescent cells targets p16^Ink4a^‐positive microglia and reduces markers of neuronal senescence in the CA3 region of the hippocampus. (a) Micrographs show immuno‐RNA‐*ISH* staining against p16^Ink4a^ mRNA and microglial marker Iba1 on brain sections in hippocampal region of young and old animals (blue = DAPI, green = iba1, red = p16 (in distinct foci), scale bars = 10 μm). Quantification of (b) fraction of p16^Ink4a^ ‐positive microglia and (c) the total number of microglia in murine hippocampal sections. Quantification of fraction of p16^Ink4a^‐positive (d) oligodendrocytes and (e) neurons of CA3 region. (f) Lamin B1 staining Micrographs showing Lamin B1 staining of brain sections in vehicle‐ (v), AP20187‐ (AP), or Dasatinib and Quercetin (D + Q)‐treated animals (blue = DAPI, green = Lamin B1, scale bar 50 μm, white arrows mark Lamin B1‐negative neurons) (g) Quantification of Lamin B1‐negative neurons. (h) Representative images of HMGB1 staining in the hippocampus, HMGB1 stained by NovaRed (Brown) and counterstained with Hematoxylin (Blue) (scale bar is 50 μm). Black arrows heads mark HMGB1‐negative neurons. (i) Quantification of neurons with no nuclear HMGB1 in the pyramidal layer of the CA3 region of the hippocampus. (j) Representative images of p21 RNA‐*ISH* in the CA3 hippocampus of *INK*‐*ATTAC* animals with the indicated treatments. (k) Quantification of p21 mRNA‐positive cells in the pyramidal layer of the CA3 hippocampal region (red = p21 mRNA, blue = DAPI, scale bar 50 μm). White arrows heads mark p21‐positive neurons. All data are mean ± SEM with *n* = 5–9 for A‐E ad *n* = 6–10 for F‐K. *p < 0.05, **p < 0.01

To address these issues and to assess the identity of the cell types which contribute to the age‐related memory loss and are targeted by senolytic interventions, we performed an assessment of p16^Ink4a^ expression in situ using RNA‐*ISH* in combination with immunostaining for markers of microglia, oligodendrocytes, and neurons. We found the number of p16^Ink4a^‐positive microglia (Iba1^+^) increases significantly in the hippocampus of aged mice (p=0.0046) (Figure 3a,b) and that in AP‐, but not D + Q‐treated mice, there was a significant reduction in the frequency of p16^Ink4a^‐positive microglia (p=0.048, p>0.05) (Figure 3b) in comparison to vehicle treated animals. We also observed that the total number of microglia increased with aging (p<0.001) and was significantly reduced upon AP (p=0.0055), but not D + Q (p=>0.05) treatment (Figure 3c). In contrast to microglia, neither oligodendrocytes nor neurons showed any changes in p16^Ink4a^ expression as a function of age or treatment (p=0.147, p=0.453) (Figure 3d,e). We did not find any significant differences in proliferation marker PCNA in microglia and oligodendrocytes in any of the groups (not shown).

Markers of cellular senescence have been shown to increase in neurons during aging (Jurk et al., 2012). To determine if other markers of neuronal senescence, apart from p16^Ink4a^, were affected by treatment with AP or D + Q, we analyzed the expression of established senescent markers Lamin B1 (LMNB1) (Shah et al., 2013), High Mobility Group Box 1 (HMGB1) (Davalos et al., 2013), and p21 (d'Adda di Fagagna, 2008) in hippocampal neurons of aged mice. We observed an age‐dependent increase in LMNB1‐negative and HMGB1‐negative neurons with aging and that treatment with AP or D + Q resulted in a significant decrease in these markers within CA3 (Figure 3f‐i), but not CA1 (Figure S3B‐E).

To measure p21 expression in hippocampal neurons, we used RNA‐*ISH*. P21‐positive neurons increased with age and were significantly reduced by AP but not D + Q treatment (Figure 3j,k; Figure S3F,G).

### 2.4 Clearance of senescent cells reduces hippocampal microglial activation, age‐related brain inflammation, and infiltration of immune cells

**FIGURE 4:**
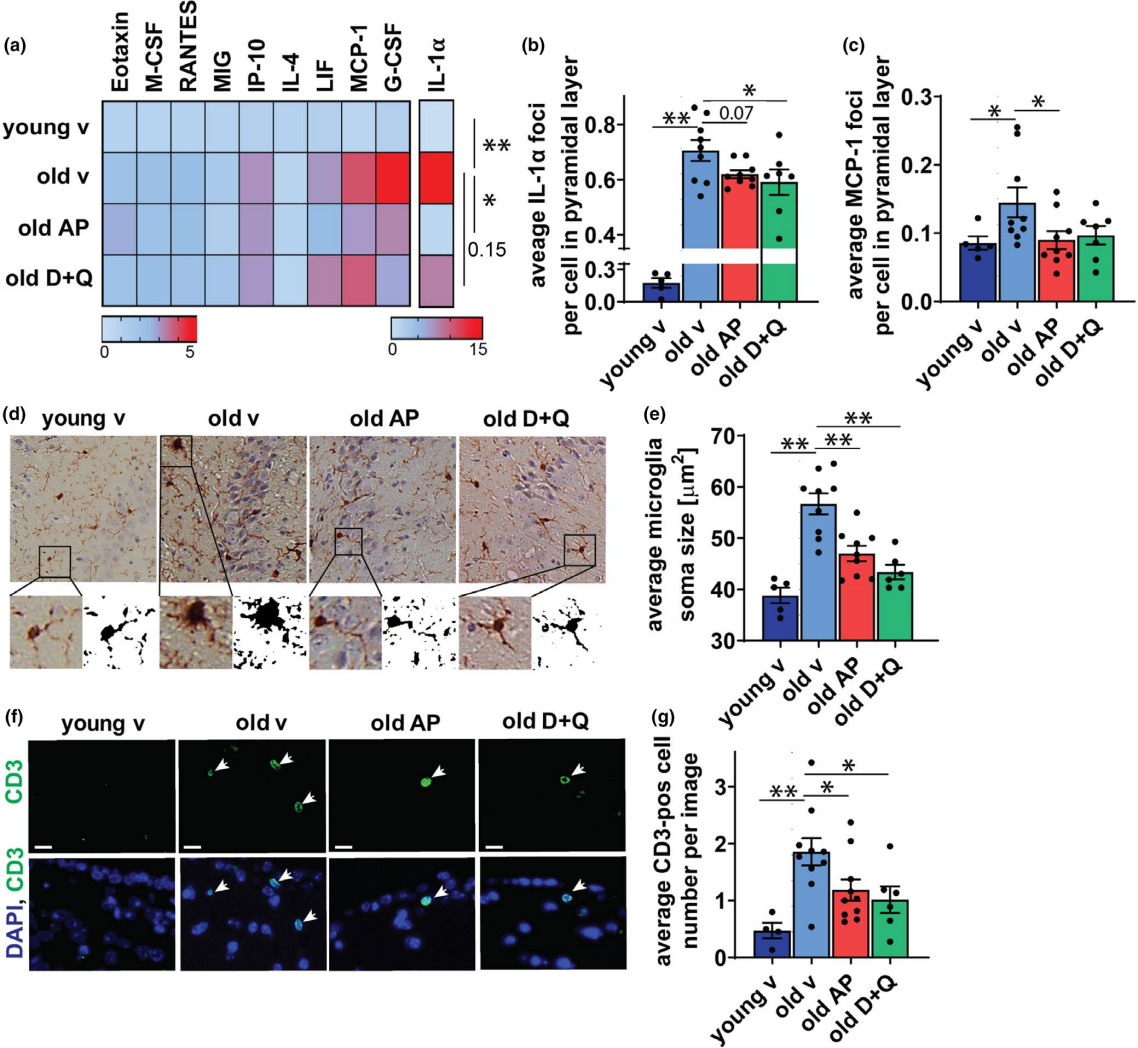
Treatments targeting senescent cells reduce brain inflammation. (a) Changes in cytokine protein levels [fold change] in the brains of young mice and old mice treated with vehicle (v), AP20187 (AP), or Dasatinib and Quercetin (D + Q). Cytokine concentrations significantly (*p* < 0.05) change between young and old brains during aging. Only cytokines that showed significant changes between young and old are shown. (b) Graph shows mean number of IL‐1α mRNA foci *per* cell of CA3 pyramidal layer. (c) Graph shows mean number of MCP‐1 mRNA foci *per* cell in the CA3 pyramidal layer. (d) Representative images of immunohistochemical staining using antibodies against Iba‐1 protein. (e) Quantification of the average soma size of microglia in the CA3 hippocampal region. (f) Representative images of CD3 immunofluorescence in the area of lateral ventricle (LV) proximal to hippocampus of young vehicle‐treated and old mice treated with vehicle, AP, or D + Q (scale bars 10 μm). (g) Quantification of the mean number of CD3^+^ cells around LV of young and old mice with or without treatment (CD3 is green, DAPI is blue). All data are mean ± SEM *n* = 4–10. *p < 0.05, **p < 0.01

An increase in inflammation is one of the most common and well‐described markers associated with cognitive dysfunction, neurodegeneration, and brain aging (Bodles & Barger, 2004; Currais et al., 2017; Yin et al., 2016). To assess the effects of aging and senescent cell clearance on brain inflammation, we performed a cytokine array analysis on whole‐brain homogenates from young and aged *INK*‐*ATTAC* mice subjected or not to senolytic treatment. Of the 32 cytokines screened, 29 were found at detectable concentrations in whole‐brain homogenates (Figure S4A) and 10 were significantly different between young and old mice (Figure 4a). We found that IP‐10, MCP‐1, and RANTES were significantly increased (Figure S4A). AP or D + Q treatment did not result in a statistically significant change of any of the individual cytokines (Figure S4A). However, a two‐way ANOVA analysis revealed that age‐associated changes in cytokine concentrations were significantly (*p* = 0.026) alleviated by AP treatment, while there was no significant effect of D + Q treatment (*p* = 0.15) (Figure 4a). Since our data are from whole‐brain homogenates and therefore may not reflect changes in cytokine expression in specific regions of the brain, we sought to characterize inflammation in the hippocampus. For that, we first quantified IL‐1α expression, an upstream regulator of the SASP (Orjalo et al., 2009), in cells of CA3 hippocampal pyramidal layer *in situ* using RNA‐*ISH* (Figure 4b). We found that the average number of IL‐1α mRNA foci increases significantly during aging in the CA3 region (p<0.0001) (Figure 4b). Importantly, this phenotype was significantly alleviated by treatment with D+Q( p=0.0278)), while AP treatment showed no significant difference (p=0.07)(Figure 4b). RNA‐*ISH* quantification of MCP‐1 mRNA expression in the hippocampus showed no age‐related increase as well as no significant reduction upon AP treatment (Figure 4c).

Another feature of brain inflammaging is microglial activation, which is characterized by increased soma size and is observed during aging and neurodegenerative diseases (Luo et al., 2010; Norden & Godbout, 2013). We found that microglia in the hippocampi of aged mice have significantly increased sizes of soma compared to their young counterparts and sizes of soma are significantly reduced by both AP and D + Q treatments (Figure 4d,e and Figure S4B).

It was recently reported that T cells infiltrate into the periventricular brain regions of old mice (Dulken et al., 2019; Ritzel et al., 2016). This process has been linked to brain inflammation and neurodegeneration (Buckwalter et al., 2006; Dulken et al., 2019). As the SASP has been shown to recruit immune cells, including T cells (Kang et al., 2011; Sagiv & Krizhanovsky, 2013), we hypothesized that targeting senescent cells could impact the number of infiltrating immune cells in the brains of aged mice. Immunostaining against CD3 revealed the presence of T cells in the areas surrounding lateral ventricle proximal to the hippocampus (Figure 4f). The number of infiltrating T cells was significantly increased in the brains of old vehicle‐treated mice and was reduced following treatments with AP and D + Q (Figure 4f,g). Our observations provide evidence suggesting that clearance of senescent cells may be a promising strategy to rejuvenate the aging brain.

## 3 DISCUSSION

Age‐associated cognitive impairment has been attributed to multiple molecular processes including chronic inflammation, impaired autophagy, macromolecular damage, and mitochondrial dysfunction (Bodles & Barger, 2004; Chow & Herrup, 2015; Head, 2009; Paradies et al., 2011; Todorova & Blokland, 2017; Yin et al., 2016). Recent data have shown that senescence plays a key role in the brain. Clearance of senescent cells has been shown to improve phenotypes in mouse models of Parkinson's disease (Chinta et al., 2018), tau‐dependent neurodegenerative diseases (Bussian et al., 2018; Musi et al., 2018), β‐amyloid‐related neurodegeneration (Zhang et al., 2019), and neuropsychiatric disorders (Ogrodnik et al., 2018). However, whether senescence impacts cognitive impairment observed during the aging process has not been fully investigated.

To address the relationship between senescent cell accumulation and brain aging, we designed studies to eliminate highly p16^Ink4a^‐expressing cells through pharmacogenetic (*INK*‐*ATTAC* mouse model) and senescent cells through pharmacologic (senolytic drugs: Dasatinib and Quercetin (D + Q)) approaches. We hypothesized that removal of senescent cells may have beneficial consequences for the aging brain and murine cognition. This idea was tested in aged *INK*‐*ATTAC* mice with initiation of treatments at 25–29 months of age.

We included young mice to test for potential age‐independent effects of the interventions. For example, side effects arising from continuous administration of Dasatinib to cancer patients (as opposed to the intermittent administration when it is used as a senolytic) have been reported (Montani et al., 2012). Quercetin, while having senolytic properties when administered intermittently, can also act as an anti‐oxidant when administered continuously (Oliveira et al., 2016). Also, since multiple cell types other than senescent cells can have high p16^Ink4a^ expression, such as activated macrophages or foam cells (Hall et al., 2016, 2017), AP may have off‐target effects by eliminating non‐senescent cells in *INK*‐*ATTAC* mice. For all the interventions in old mice, we observed significantly better cognitive function, decreased markers of senescence, p16^Ink4a^, High Mobility Group Box 1 (HMGB1) and Lamin B1 (LMNB1), and a mild reduction in markers of inflammation associated with decreased microglial activation and numbers of brain‐residing T cells. These changes were not seen after treating young mice. The post‐treatment effect of the interventions on cognition remained significant even after the data were normalized to baseline measurements. This excludes the possibility of a survival effect contributing to the improvement, indicating the performance of individuals changed, rather than being a result of selection for survival of better‐performing mice. In contrast, a previously published study reported no effect of senescent cell clearance on memory of *INK*‐*ATTAC* mice (Baker et al., 2016). We believe that the apparent discrepancies between both studies are related to fundamental differences in the experimental design. In Baker et al. (2016), the authors used the novel object investigation test to assess memory. The results of this test are likely to be influenced by age‐related changes commonly observed in aging mice, such as blindness and impaired sense of smell. For that reason, in our study, we used the Stone T‐maze test, which is not affected by visual or olfactory impairment (Pistell & Ingram, 2010; Pistell et al., 2012). Additionally, the mice used in the study by Baker et al. (2016) were started on AP treatment to reduce highly p16^Ink4a^‐expressing cells at a relatively young age (12 months), while our mice started treatments at 25–29 months of age. Based on previous data, senescent cell markers only increase at later ages (Jurk et al., 2012). It should be pointed out that, although Baker et al. (2016) reported no significant differences in the novel object investigation test, their data show a higher score in memory testing of both male and female mice treated with AP (Baker et al., 2016).

Our experimental approach has some limitations, including the fact that the senolytic interventions used in this study target the whole body. In this respect, we are not able to determine whether the improvement in cognition is a result of senescent cell clearance in the central nervous system or in peripheral organs (including immune cells). However, we should point out that all agents used in this study have been shown to cross blood–brain barrier (Ishisaka et al., 2011; Porkka et al., 2008), which is consistent with previously published studies reporting a reduction of cellular senescence in the murine brain upon senolytic treatment (Bussian et al., 2018; Chinta et al., 2018; Zhang et al., 2019). We also acknowledge that while our data supports a role for senescence in the age‐dependent cognitive decline, additional tests examining cognitive function are needed to further validate this study's findings.

During aging, it has been reported that senescent cell markers increase in various cell types such as neurons, astrocytes, microglia, and ependymal cells (Bussian et al., 2018; Chinta et al., 2018; Jurk et al., 2012; Ogrodnik et al., 2018), among others. Here, we confirmed by single‐cell and ‐nuclei RNA‐seq that p16^Ink4a^ expression increases with age in different cell populations, however, it is more predominant in microglia and oligodendrocytes. Importantly, we found that both pharmacogenetic and pharmacological interventions targeting senescent cells are only effective at removing p16^Ink4a^‐positive microglia but not neurons or oligodendrocytes, which is consistent with previous reports in the context of Tau‐dependent neurodegeneration (Bussian et al., 2018).

Additionally, we found that neurons of CA3 regions in the hippocampus have other markers of senescence, such as reduction in LMNB1 and nuclear exclusion of HMGB1, which are well‐established markers of senescence (Davalos et al., 2013; Shimi et al., 2011). It should be noted that nuclear exclusion of HMGB1 may also occur as a consequence of stress, independently of senescence. While we focused on neurons in the hippocampus, a more detailed characterization of senescence in different cell types and brain regions should be conducted. While we speculate that reduction of senescence markers in neurons may be an indirect consequence of elimination of p16^Ink4a^ expressing senescent microglia, it remains a possibility that D + Q is directly targeting SCAPs in neurons. Cell type‐specific models of senescent cell clearance would greatly help our understanding of the contribution of different types of cells to the observed phenotypes.

Finally, the interventions to clear senescent cells we used in this study might have different activities or even target different subsets of senescent cells. In the case of *INK*‐*ATTAC* mice, as stated above not all senescent cells have high p16^Ink4a^ expression and not all cells with high p16^Ink4a^ expression are senescent. In *INK*‐*ATTAC* mice, AP kills cells based on high expression of p16^Ink4a^, potentially including activated immune cells such as macrophages (Hall et al., 2016, 2017). Thus, we cannot exclude the possibility that AP affects infiltrating immune cells directly in *INK*‐*ATTAC* mice instead of through indirect effects involving reduced immune cell attraction by senescent cells. D + Q treatment does not specifically target p16^Ink4a^‐expressing cells and has been shown to not eliminate activated macrophages (Palmer et al., 2019; Xu et al., 2018). While D + Q can have a broad spectrum of activities when present continuously, including tyrosine kinase inhibition (Dasatinib), HIF‐1 alpha induction, and reactive oxygen species scavenging (Quercetin) (Steinberg, 2007; Triantafyllou et al., 2008; Zhu et al., 2015), both of these senolytic drugs have short elimination half‐lives (<11 hours). Thus, because D + Q was only administered intermittently, not continuously, such off‐target effects of Dasatinib or Quercetin that depend on continuous presence or the drugs to inhibit an enzyme, occupy a receptor, or target a biochemical pathway are unlikely. While our data supports the hypothesis that D + Q induces apoptosis in senescent microglia, it remains to be established if it is directly targeting its SCAPs.

Mechanistically, it is still not clear how senescence impacts brain aging. It has been speculated that chronic inflammation driven by senescence can negatively influence neurogenesis (Chinta et al., 2018; Ogrodnik et al., 2018), spread senescence to surrounding cells (Acosta et al., 2013; Nelson et al., 2012; Xu et al., 2018), and contribute to neurodegeneration (Bussian et al., 2018; Chinta et al., 2018; Musi et al., 2018; Zhang et al., 2019). Accordingly, we observed that senolytic treatment decreased expression of known SASP components and markers of inflammation. We should point out, however, that the effects were relatively mild. Thus, it is possible that the strategies employed here were not effective in the removal of all senescent cells; or alternatively that senescent cells in the brain are not the major contributors to age‐dependent inflammation. Thus, a more comprehensive analysis of senescent cell markers in different regions of the brain following interventions is warranted. Despite these caveats, we are encouraged by the consistent effect of two different interventions that can target senescent cells on cognitive function and the phenotype of brain aging in old mice.

On the basis of our findings, we speculate that cellular senescence contributes to the process of brain aging and to driving age‐related cognitive impairment. Our study provides proof‐of‐concept evidence indicating that senotherapies (Sikora et al., 2019) can be tested as a therapeutic approach for rejuvenating the brain and improving memory function in older people.

## 4 METHODS

### 4.1 Animals

Experimental procedures were approved by the Institutional Animal Care and Use Committee at Mayo Clinic (protocol A26415). *INK*‐*ATTAC* ^+/−^ transgenic mice were generated and genotyped as previously described (Baker et al., 2011) based on experimental strategies devised by J.L.K., T.T., J. van Deursen, and D. Baker at Mayo Clinic.

Mice were housed 2–5 *per* cage, at 22 +/‐ 0.5°C on a 12–12‐h day‐night cycle and provided food and water *ad libitum*. Mice were allowed to age without any interventions until 4 and 25–29 months of age, when they were randomly assigned into AP20187, D + Q, or vehicle treatment groups. Mice were injected intraperitoneal (i.p.) with AP20187 or vehicle and were gavaged with Dasatinib and Quercetin in combination or with vehicle (i.e., each mouse received both gavage and an i.p. injection with at least one of them containing vehicle). Drugs or vehicle were administered at 9‐11am for 3 consecutive days *per* week, every two weeks for the total period of 8 weeks. Concentrations of drugs and vehicle composition are listed in Table 1.

**TABLE 1:** Drugs used in the mouse studies

	Dose	Drugs supplier	Vehicle	Route
AP20187	10 mg/kg body weight	ARIAD Pharmaceuticals	4% ethanol, 10% PEG‐400, and 2% Tween‐20 in distilled water	i.p. injection
Dasatinib and quercetin (D + Q)	Dasatinib 5 mg/kg body weight Quercetin 10 mg/kg body weight	LC Laboratories (Dasatinib) Sigma (Quercetin)	60% Phosal 10% ethanol 30% PEG‐400	Oral gavage

### 4.2 Single‐cell and single‐nuclei RNA sequencing

For single‐cell RNA sequencing, the hippocampus was dissected from the brains of 4‐month and 24‐month C57BL/6 mice. There were a total of 4 animals used for each age group. For the 24‐month‐old mice, the hippocampi from 2 mice were pooled together into 1 sample, and the other 2 mice were pooled into another sample. The same pooling scheme was used for the 4‐month‐old mice. Once the hippocampi were dissected and pooled together, the single‐cell suspensions were created using the Miltenyi Biotech gentleMACS dissociator and the papain based Neural Tissue Dissociation Kit according to the manufacturer's instructions. Red Blood Cells were removed using the Miltenyi Red Blood Cell Lysis Buffer. For each sample, we targeted 5,000 cells to load onto the 10x chromium device using Version 2 chemistry. Samples were sequenced at GeneWiz where we targeted 50,000 reads per cell on an illumina HiSeq device. We performed two rounds of sequencing using these parameters for each sample.

The same sample pooling scheme as the single‐cell RNA sequencing that was described above was utilized for single‐nuclei RNA sequencing, although different animals were used for the dissections. Nuclei were isolated from minced hippocampi tissue using the Nuclei PURE Prep Nuclei Isolation Kit with a Dounce B homogenizer. Samples were subjected to a sucrose gradient, and nuclei were further purified and counted. We targeted 5000 nuclei per sampled to load onto a 10x Chromium chip using VD(J) chemistry. We targeted 50,000 sequence reads per nuclei on an illumina HiSeq device. Only one round of sequencing was performed for single‐nuclei data.

### 4.3 Single‐cell and single‐nuclei RNA‐seq Analysis

Single‐cell RNA‐seq fastq files were aligned to the mm10 genome using CellRanger version 2.1.0. Meanwhile, single‐nuclei RNA‐seq fastq files were aligned using CellRanger Version 3.0.2 to a custom‐made pre‐mRNA reference which was created according to 10x Genomics instructions.

Downstream analysis was conducted with the Seurat bioconductor package (Version 3.1.4). Data from each sample were normalized using the SC‐Transform function in Seurat. Samples for the single cell were combined together using the IntegrateData function and single‐nuclei data were combined in the same manner. Cell types were identified with a custom‐made list of gene markers.

Data under the “RNA” assay of the Seurat object was Log‐normalized using the NormalizeData function and then scaled using the ScaleData function. This gave our single‐cell and single‐nuclei Seurat objects 3 different “slots” under the RNA assay. One slot was the raw counts, the second was the log‐normalized data, and the third was the scaled data. Heatmaps were generated with scaled data, which can give negative values for some cells. UMAP projections were calculated with the first 30 principal components of the log‐normalized data. We used the FindMarkers function using MAST methodology for computing the log‐fold changes for each gene between old and young cells and their corresponding fdr‐values (Finak et al., 2015). Violin plots were generated with the normalized data and plotted on a log scale.

For single‐cell and single‐nuclei data, p16 positive cells were identified by the presence of at least one Cdkn2a transcript. SASP lists were obtained from De Cecco et al. (2019).

### 4.4 Body composition

Lean and fat mass of individual mice were quantified by quantitative nuclear magnetic resonance using an EchoMRI analyzer (Houston, TX) and normalized to body weight. Un‐anesthetized animals were placed in a plastic tube that was introduced into the EchoMRI instrument. Body composition, consisting of fat mass and lean mass, was generated in approximately 90 s *per* animal.

### 4.5 Rotarod

The Rotarod performance test evaluates mouse balance and motor coordination. Mice where brought to the test room a day before the testing and habituated overnight. For baseline tests, mice were trained on the Rotarod (3375‐M5; TSE systems) first for three consecutive days. On each training day mice were placed (having their back turned toward the experimenter) on the 4.0 cm diameter rotating rod. Mice were allowed to stay on the rod for 200 s with its speed for each of three days at 4 rpm, 6 rpm, and 8 rpm, respectively. If the mouse fell during training, it was put back on the rod. On the test day, mice were placed on the rod the same way as on the training days. The Rotarod was then started at 4 rpm and accelerated to 40 rpm over a 5 min interval. Mice were allowed three trials and the times to fall and the speeds at which mice fell were recorded for each mouse at all three attempts. Two months after baseline measurements, mice were tested again. Mice were habituated to the test room overnight and treated the same way as for the baseline tests. Mice had a 1 day training session with rod's speed being 6 rpm. On the next day, mice were tested in 3 consecutive trials, consistent with the baseline measurements. The average of three trials normalized to the baseline was taken as an indicator of mouse balance and motor coordination.

### 4.6 Stone T‐maze

A water‐motivated version of the Stone T‐maze was used (custom‐made by Mayo Clinic workshop) to measure parameters of cognition. A straight run (for pre‐training) or Stone T‐maze were placed into a steel pan filled with water to a depth of approximately 3 cm, which covered half the height of the interior walls of the maze. The ceiling of both the straight run and maze were covered with clear acrylic to prevent mice from rearing out of the water. These dimensions created a situation that enables the mice to maintain contact with the floor while maintaining their heads above water. The mice were placed into a start box and were pushed into the maze using a sliding panel that is moved using a rod extending out of the rear wall. At the end of the straight run or maze there was a goal box that contains a ramp to a dry floor that allows the mice to escape from the water upon successful completion of the straight run or maze. On day one, mice underwent straight run training to establish the concept that moving forward allows them to escape the water by reaching a water‐free goal box. Successful completion of this phase requires the mice to reach the goal box in 10 s or faster, in 6 out of 9 trials. Mice that did not reach this criterion were excluded from further testing. Maze training commenced the following day. Mice completed 9 maze acquisition trials in a single day. All mice performed one trial before performing the next one. Runs using between 6 and 8 mice resulted in inter‐trial intervals (ITI) of approximately 5–12 minutes. During ITI, mice were placed in a holding cage containing a dry towel that was additionally heated by a red heat lamp. Primary measures of learning and memory were the latency to reach the goal box and the numbers of errors committed. An error was defined as complete entry of the mouse's head or the whole body into an incorrect path. During the acquisition phase, if any mouse failed to reach the goal box within 5 min, the trial was terminated and scored as a failure. Any mouse receiving 3 failures was removed from further trials. During the straight run training all tested mice reached the goals in less than 10 s at least 6 times, and no mice received more than 2 failures in the Stone maze, thus none of the mice was excluded from the studies. Two‐way analysis of variance (ANOVA) was used to analyze the data.

### 4.7 Brain cytokine array

Brain homogenates were prepared by cryogenic grinding of the one hemisphere of each brain. Resulting powder was dissolved in RIPA buffer (0.1 M Tris buffer, 0.5% TRITON, protease inhibitor cocktail (Thermofisher) 1:100, pH 7.4). The homogenate was then incubated on ice for 30 min, centrifuged (30 min at 4°C, 16.1 rcf) and the supernatant was collected. Protein concentrations were determined by Bradford Assay (Biorad) and 85 μl of sample volume at 10 μg/μl was sent for cytokine array (Eve Technologies). Brain levels of cytokines: Eotaxin, G‐CSF, IFN‐γ, IL‐1 α, IL‐1β, IL‐2, IL‐4, IL‐5, IL‐6, IL‐7, IL‐9, IL‐10, IL‐12, IL‐15, IL‐17, KC/CXCL1, LIF, MCP‐1, M‐CSF, MIG, MIP‐1α, MIP‐1β, RANTES, MIP‐2, TNF‐α, and VEGF were determined using a Multiplexing LASER Bead Assay (Eve Technologies; Canada). To select cytokines related to brain aging, *t* tests were performed on brain cytokine concentrations from young and old vehicle‐treated mice. The treatment effect was determined by two‐way ANOVA analysis of cytokines, with those cytokine that changed significantly during aging identified by comparing concentrations of these cytokines between old vehicle‐ and old AP‐treated or between old vehicle‐ and old D + Q‐treated mice.

### 4.8 Histochemistry, immunofluorescence, and immuno‐FISH

Paraffin sections were deparaffinized with Histoclear (National Diagnostics) and ethanol (Fisher Chemical). Antigen was retrieved by incubation in 0.01 M citrate buffer (pH 6.0) at 95 °C for 20 min. For immunohistochemistry (IHC), slides were incubated in 0.9% H_2_O_2_ for 30 min. Afterward slides were placed in blocking buffer (normal goat serum 1:60 in PBS/BSA, #S‐1000; Vector Laboratories) for 60–120 min at room temperature. For Iba1 and HMGB1 IHC stainings, tissues were further blocked with Avidin/Biotin (Vector Laboratories, no. SP‐2001) for 15 min each. Sections were incubated with primary antibodies (Table 2) at 4°C overnight. Slides were washed three times with PBS and incubated for 60 min with secondary antibody (Table 2). For IHC staining, antibodies were detected using a rabbit peroxidase ABC Kit (no. PK‐6101; Vector Lab) according to the manufacturer's instructions and the substrate was developed using NovaRed (no. SK‐4800; Vector Lab) and sections were counterstained with hematoxylin. Slides for IF were mounted using Antifade Mounting Medium with DAPI (Vector Laboratories, no. H‐1200‐10) and slides for IHC were mounted using DPX.

**TABLE 2:** Antibodies used for immunostaining

Name of an antigen	Company producing primary antibody and catalogue number	Primary antibody: origin and concentration	Secondary antibody: origin and concentration	Tertiary antibody or developing system
CD3	AbD Serotec, #MCA1477	Rat, 1:100	Anti‐rat, biotinylated, Goat, 1:200	DSC‐fluorescein (Vector Lab)
Lamin B1	Abcam, ab16048	Rabbit, 1:500	Anti‐rabbit, Alexa 488, Goat, 1:1000	
HMGB1	Abcam, ab18256	Rabbit, 1:500	Anti‐rabbit, biotinylated, Goat, 1:200	Horseradish peroxidase ABC kit, NovaRed (Vector Lab)
NeuN/FoxO	Abcam, ab104224	Mouse, 1:500	Anti‐mouse, Alexa 647, Goat, 1:500	
Gfap	Synaptic systems, Cat. 173004	Guinea pig, 1:500	Anti‐guinea pig, Alexa 647, Goat, 1:500	
Iba1	Abcam, ab178846	Rabbit, 1:2000 (IHC) 1:500 (IF)	Anti‐rabbit, biotinylated, Goat, 1:200 (IHC) or 1:1000 AlexaFluor 488 (IF)	Horseradish peroxidase ABC kit, NovaRed (Vector Lab)
Olig2	Millipore, AB9610	Rabbit, 1:250	Anti‐rabbit, Alexa 647, Goat, 1:1000	

### 4.9 Iba1^+^ cell density and soma size quantifications

Scans of the hippocampus were imaged using a Leica DMi‐8 in brightfield on IHC sections stained for Iba1^+^. The hippocampus was then subdivided into CA3 and CA1 regions in ImageJ. The number of Iba1+ cells in region, and the area of each region were recorded. Cell numbers were expressed as number of Iba1^+^ cells per mm (Camandola & Mattson, 2017) in a 10 µM thick section. The area of the soma of Iba1+ cells (excluding processes) were manually traced and measured in ImageJ.

### 4.10 RNA‐ISH

RNA‐ISH was performed according to RNAscope protocol from Advanced Cell Diagnostics (ACD). Paraffin sections were deparaffinized with Histoclear and rehydrated in 100% ethanol (EtOH). Sections were allowed to air dry and then incubated with H_2_O_2_ for 10 min at room temperature followed by another 2 washes in H_2_O. Sections were placed in hot 1X retrieval solution and heated for 15 min. After washes in H_2_O and 100% EtOH, sections were air dried. Sections were treated with protease plus for 30 min at 40°C, washed with H_2_O, and incubated with target probes: p21 (#408551), p16 (#411011), IL1α (#440391) or MCP‐1 (#311791) for 2 h at 40°C. Next, slides were washed with H2O followed by incubation with AMP1 (30 min at 40°C) and next washed with wash buffer (WB) and AMP2 (15 min at 40°C), WB and AMP3 (30 min at 40°C), WB and AMP4 (15 min at 40°C), WB and AMP5 (30 min at RT) and WB, and, finally, AMP6 (15 min at RT). Lastly, an RNAscope 2.5 HD Reagent kit‐RED was used for chromogenic labelling. Sections were washed in H_2_O five times followed by 2 TBS washes for 5 min each, and were then mounted using ProLong Gold mounting media containing DAPI.

For analysis of p16, sections were co‐stained with antibodies for Olig2 and Iba1. Briefly, following chromogenic labelling for cytokines as described above, sections were washed 3 times in TBS for 5 min each followed by blocking in 0.1%BSA in PBS for 30 min at RT. Sections were incubated overnight with primary antibodies at 4°C. Next, sections were washed 3 times in TBS for 5 min each followed by secondary antibody incubation for 1 h at RT. After 3 TBS washes sections were mounted using ProLong Gold mounting media containing DAPI. For p16 and p21 quantifications, the number of cells having one or more foci was counted. For cytokine (MCP1 and IL1α) expression experiments data were analyzed by quantifying the number of foci in each cell of CA1 and CA3 pyramidal cell layer.

### 4.11 RT‐PCR

Hippocampal area RNA was extracted from 24–25‐month‐old mice using TRIzol. The RNA concentration was determined by Nanodrop. RNA was reverse‐transcribed to cDNA using High Capacity RNA to cDNA kit as per manufacturer's instructions (Thermo Fisher Scientific). TaqMan fast advanced master mix was used for real‐time PCR. TATA‐binding protein (TBP) was used as a housekeeping gene. Probes and primers used: TBP and p16^Ink4a^.

### 4.12 Statistical analysis

Data are presented as mean ± SEM for all data. All data were assessed for normality using D'Agostino's K‐squared test. F test was used to compare variances. Contingency analysis for an overall survival in the mouse studies was done using two‐sided Chi‐square test. Single comparisons were performed using unpaired, two‐tailed Student's *t* test (for parametric data) with (for unequal variances) or without (for equal variances) Welch's correction or Mann–Whitney *U* tests (for non‐parametric data). Multiple‐group comparisons were performed using two‐way analysis of variance (ANOVA). Correlations were assessed using Pearson's (for parametric data) or Spearman's (for non‐parametric data) rank correlation tests. All the tests were performed GraphPad Prism version 8.

## CONFLICT OF INTEREST

Patents on *INK*‐*ATTAC* mice and senolytic drugs are held by Mayo Clinic. Some of these are licensed to Unity Biotechnology. J.L.K., T.T., and Y.Z. may gain financially from these patents and licenses. This research has been reviewed by the Mayo Clinic Conflict of Interest Review Board and was conducted in compliance with Mayo Clinic Conflict of Interest policies. The remaining authors declare no competing financial interests.

## AUTHOR CONTRIBUTIONS

M.O. performed the majority of experiments and gathered data; S.E., S.V., H.S. Y.Z., T.T., P.K., E.F., B.M.W., A.D.P, T.P., C.L.I., K.O.J. and A.R. performed and evaluated individual experiments; D.J. and J.L.K. with help from T.T. and J.F.P. designed and supervised the study; S.L.D. and D.B.A. did statistical evaluation of the data. D.J. prepared figures and wrote the manuscript with contributions from M.O., N.N., J.L.K., N.L., M.S. and T.v.Z.

## Supporting information

Fig S1‐S4

Video S1

Video S2

Video S3

## Data Availability

The datasets generated during and/or analyzed during the current study will available in the NCBI GEO repository after publication and the accession codes is GSE161340 (https://www.ncbi.nlm.nih.gov/geo/query/acc.cgi?acc=GSE161340). All data generated or analyzed during this study are available from the corresponding author on reasonable request.
