# Supplementary material for: Whole‐body senescent cell clearance alleviates age‐related brain inflammation and cognitive impairment in mice
Source: Aging Cell. 2021 Jan 20;20(2):e13296. doi: 10.1111/acel.13296 (PMC7884042; doi:10.1111/acel.13296)
Supplement: Supplementary file 1 — Fig S1‐S4 [file ACEL-20-e13296-s001.docx]

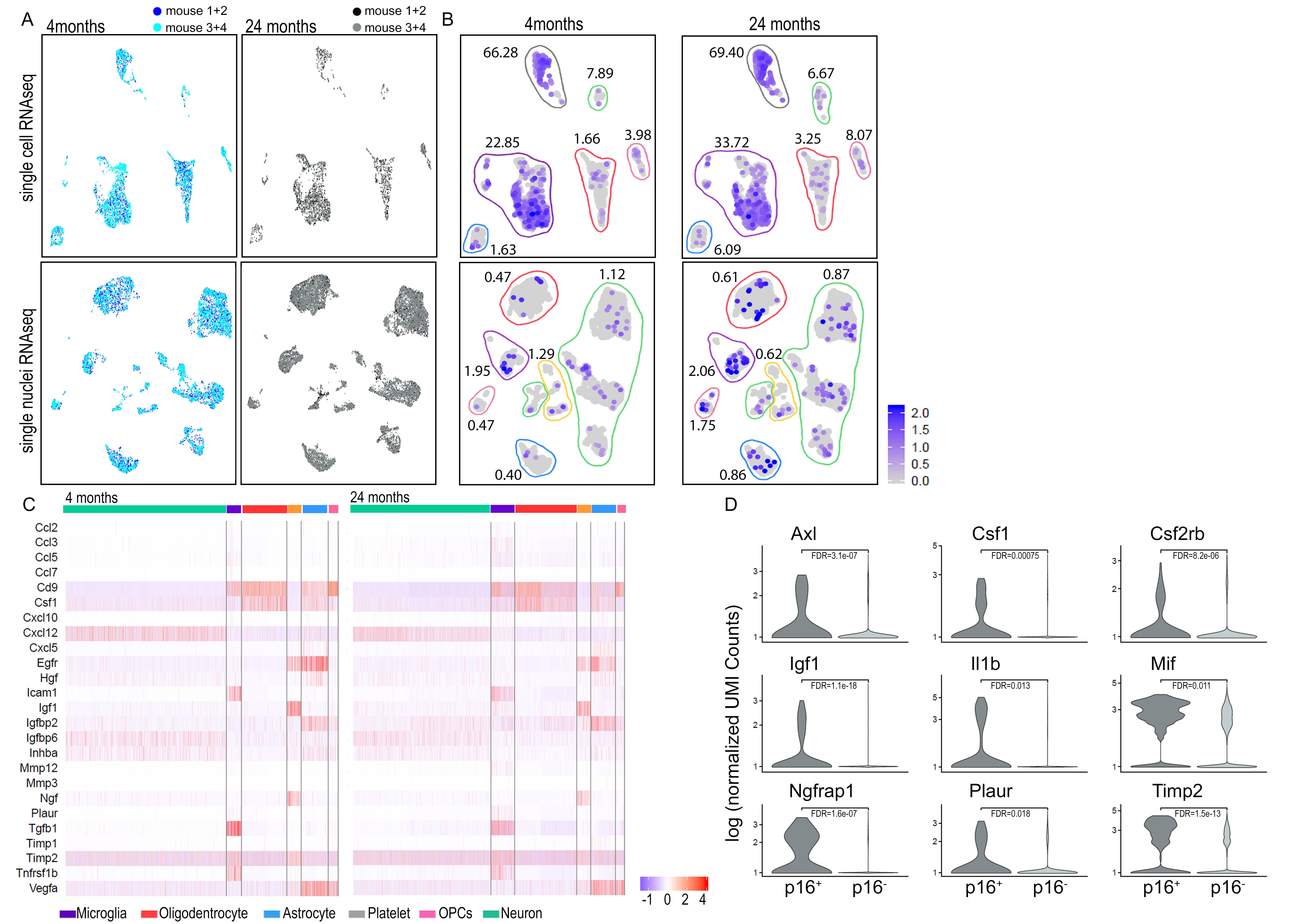


**Figure S1: Senescent cells accumulate in the hippocampus during aging.** UMAP embeddings of sc-RNA-seq (top) or sn-RNA-seq (bottom) were investigated in our datasets. **(A)** Colors represent cells per pooled sample. **(B)**. Colors represent different cell type annotations and p21 positive cells are shown in purple. **(C)** Heatmaps represent expression of cytokines in various types of brain cells identified in the single nucleus-RNA-seq dataset. Rectangles above the heatmap show color-matched cell-types consistent with graphs above. **(D)** Violin plots show expression of cytokines in microglia showing high or no expression of p16^Ink4a^. FDR was computed with Bonferroni correction using all genes in the dataset. Total of 4 mice per condition (young, old) are analyzed, while 2 mice are pooled for each experiment.

**
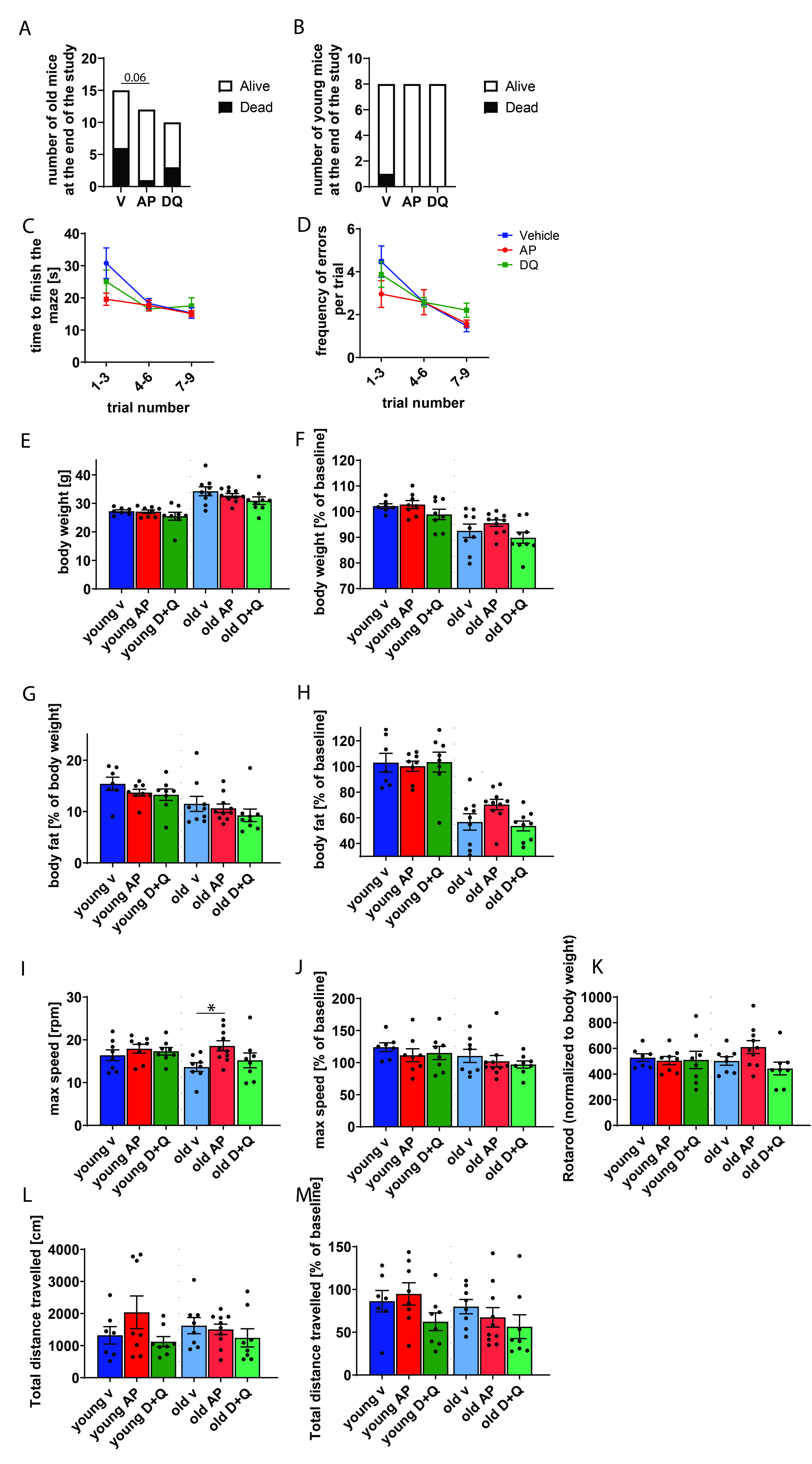
**

**Figure S2: Effect of treatments targeting senescent cells on survival, body mass and composition, Rotarod performance, and cognition. (A)** and **(B)** Number of dead and living old and young *INK-ATTAC* mice, treated with vehicle (V), AP20187 (AP), or Dasatinib and Quercetin (DQ). **(C)** The time spent to finish the maze and **(D)** the frequency of errors using the Stone T-maze were evaluated in young *INK-ATTAC* mice with or without treatments. Quantification of body weight **(E)** post-treatment and **(F)** following normalization to the baseline body mass. Body composition (shown as % of fat in total body mass) of AP-, V-, or D+Q-treated *INK-ATTAC* mice shown as **(G)** post-treatment measurements and as **(H)** parameters after normalization to the baseline. Rotarod score (maximum speed obtained by a tested mouse before falling from a rod) of the studied mice **(I)** without or **(J)** with normalization to the baseline. **(K)** Rotarod score normalized to the body mass of old *INK-ATTAC* mice. The total distance mice travel during the open field test **(L)** without or **(M)** with normalization to the baseline. All data are mean ± s.e.m. n=10-15 for A; n=8 for B; n=7-8 for young and n=9-10 for old mice for C-H; n=32 for I; n=7-8 for J-K. *p<0.05.

**
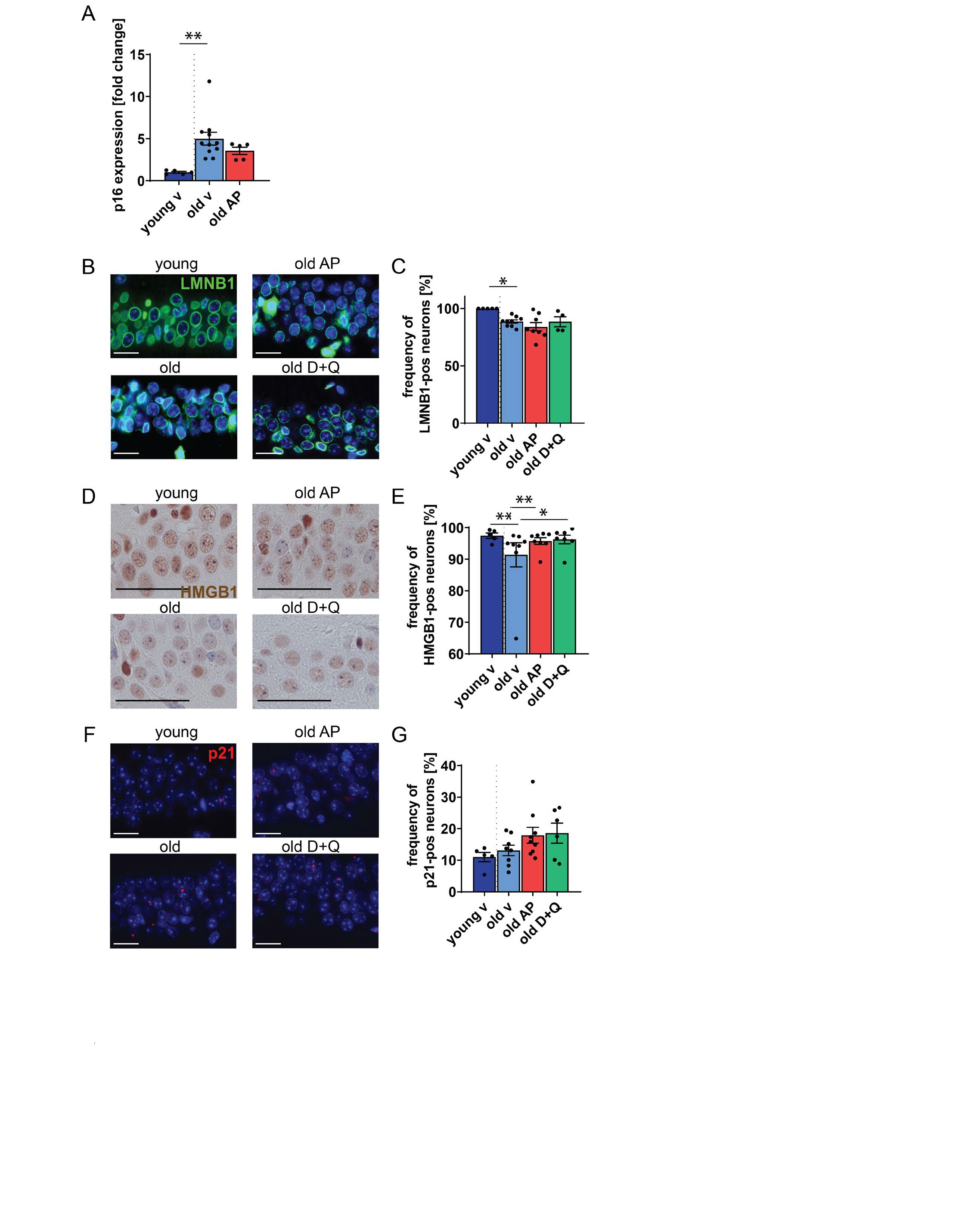
**

**Figure S3: Treatments targeting senescent cells do not affect markers of neuronal senescence in the CA1 region of the hippocampus. (A)** RT-PCR of p16 expression in hippocampus of young, old and old-AP20187-treated mice. **(B)** Micrographs showing Lamin B1 (LMNB1) staining of CA1 sections in young, old and AP20187- (AP), or Dasatinib + Quercetin (D+Q)-treated animals (blue=DAPI, green=Lamin B1, scale bar 20 μm) **(C)** Percentage of Lamin B1-negative neurons was determined using ImageJ. **(D)** Representative images of High Mobility Group Box 1 (HMGB1) staining in the hippocampus, HMGB1 stained by NovaRed (Brown) and counterstained with Haematoxylin (Blue) (scale bar = 50 μm). **(E)** Quantification of neurons with no HMGB1 in the nucleus in the pyramidal layer of the CA1 region of the hippocampus. **(F)** Representative images of p21 RNA-*ISH* staining in the CA1 hippocampus of young and old *INK-ATTAC* treated with AP or D+Q. **(G)** Quantification of p21 mRNA-positive cells in the pyramidal layer of the CA1 hippocampal region (red=p21 mRNA, blue=DAPI, scale bar 20 μm). All data are mean ± s.e.m. with n=6-10. * p<0.05, ** p<0.01.

**
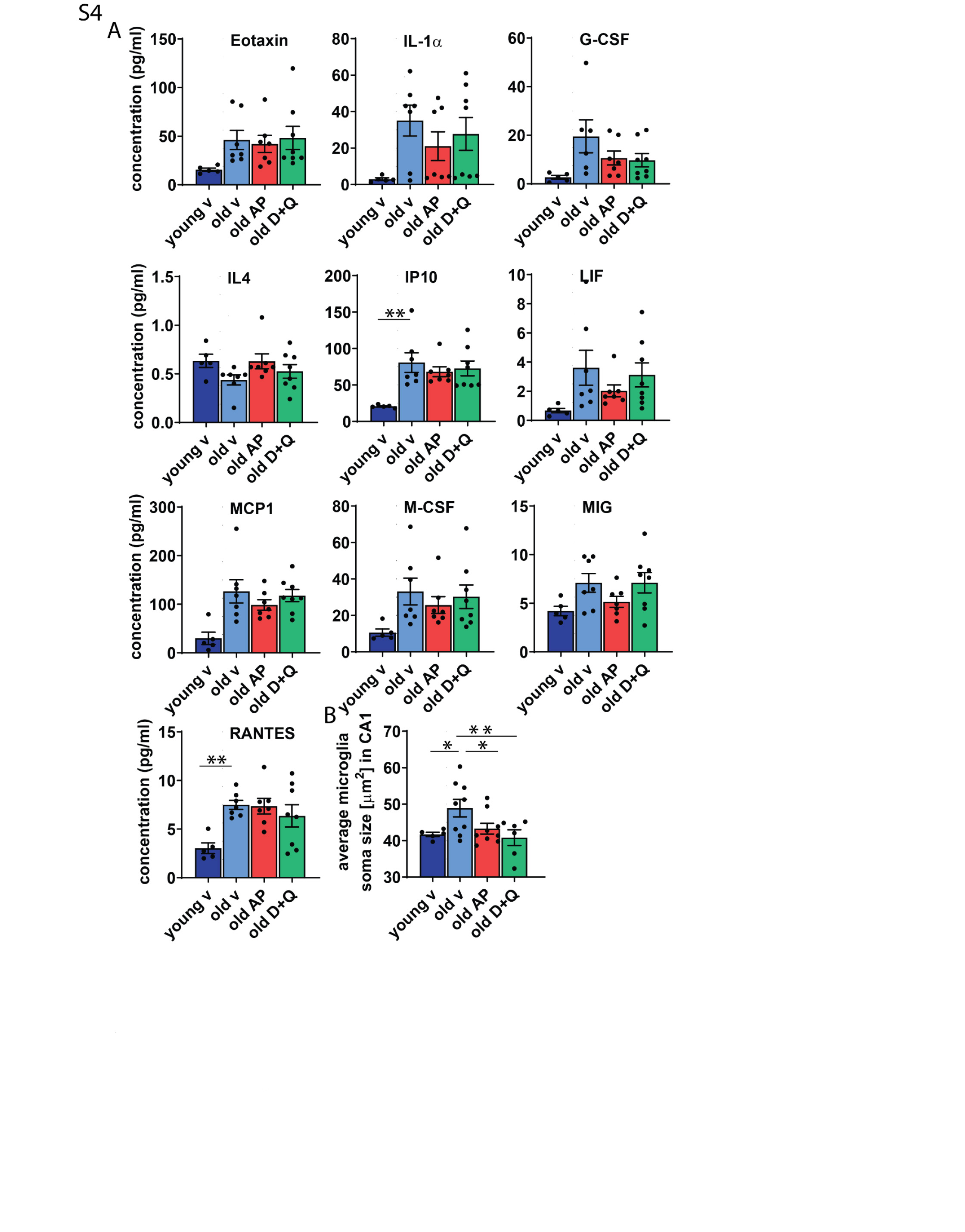
**

**Figure S4: Effects of interventions targeting senescent cells on protein expression of individual brain cytokines in whole brain homogenates. (A)** Protein levels of cytokines in the brain of vehicle- (v), AP20187- (AP), and Dasatinib and Quercetin- (D+Q) treated old and young *INK-ATTAC* mice. **(B)** Average size of the soma of microglia in the CA1 hippocampal region is shown. All data are mean ± s.e.m. n=5-8 for A, n=4-9 for B.
